# Glucagon-like peptide-1 receptor agonists use and associations with outcomes in heart failure and type 2 diabetes: data from the Swedish Heart Failure and Swedish National Diabetes Registries

**DOI:** 10.1093/ehjcvp/pvae026

**Published:** 2024-04-17

**Authors:** Markus Wallner, Mattia Emanuele Biber, Davide Stolfo, Gianfranco Sinagra, Lina Benson, Ulf Dahlström, Soffia Gudbjörnsdottir, Francesco Cosentino, Peter G M Mol, Giuseppe M C Rosano, Javed Butler, Marco Metra, Lars H Lund, Giulia Ferrannini, Gianluigi Savarese

**Affiliations:** Division of Cardiology, Department of Internal Medicine, Medical University of Graz, Graz 8010, Austria; Division of Cardiology, Department of Medicine, Karolinska Institutet, Solnavägen 1, 171 77 Solna, Stockholm, Sweden; Department of Medical Studies, University of Trieste, School of Medicine, Trieste 34149, Italy; Division of Cardiology, Department of Medicine, Karolinska Institutet, Solnavägen 1, 171 77 Solna, Stockholm, Sweden; Cardiothoracovascular Department, University of Trieste, Trieste 34149, Italy; Cardiothoracovascular Department, University of Trieste, Trieste 34149, Italy; Division of Cardiology, Department of Medicine, Karolinska Institutet, Solnavägen 1, 171 77 Solna, Stockholm, Sweden; Department of Cardiology and Department of Health, Medicine and Caring Sciences, Linköping University, Linköping 58183, Sweden; National Diabetes Registry, Centre of Registries, Gothenburg 40530, Sweden; Department of Molecular and Clinical Medicine, Institute of Medicine, University of Gothenburg, Gothenburg 40530, Sweden; Division of Cardiology, Department of Medicine, Karolinska Institutet, Solnavägen 1, 171 77 Solna, Stockholm, Sweden; Heart and Vascular and Neuro Theme, Karolinska University Hospital, Eugeniavägen 27, Norrbacka, SE-171 64 Stockholm, Sweden; Department of Clinical Pharmacy and Pharmacology, University Medical Center Groningen, University of Groningen, Groningen 9713, The Netherlands; IRCCS San Raffaele, Rome 00163, Italy; University of Mississippi, Jackson, MS 39213, USA; Baylor Scott and White Research Institute, Dallas, TX 75204, USA; Department of Medical and Surgical Specialties, Radiological Sciences, and Public Health, University of Brescia, Brescia 25121, Italy; Division of Cardiology, Department of Medicine, Karolinska Institutet, Solnavägen 1, 171 77 Solna, Stockholm, Sweden; Heart and Vascular and Neuro Theme, Karolinska University Hospital, Eugeniavägen 27, Norrbacka, SE-171 64 Stockholm, Sweden; Division of Cardiology, Department of Medicine, Karolinska Institutet, Solnavägen 1, 171 77 Solna, Stockholm, Sweden; Division of Cardiology, Department of Medicine, Karolinska Institutet, Solnavägen 1, 171 77 Solna, Stockholm, Sweden; Heart and Vascular and Neuro Theme, Karolinska University Hospital, Eugeniavägen 27, Norrbacka, SE-171 64 Stockholm, Sweden

**Keywords:** Glucagon-like peptide-1 receptor agonists, Heart failure, Type 2 diabetes, Registry, SwedeHF, Safety

## Abstract

**Aims:**

To assess the use and associations with outcomes of glucagon-like peptide-1 receptor agonists (GLP-1 RA) in a real-world population with heart failure (HF) and type 2 diabetes mellitus (T2DM).

**Methods and results:**

The Swedish HF Registry was linked with the National Diabetes Registry and other national registries. Independent predictors of GLP-1 RA use were assessed by multivariable logistic regressions and associations with outcomes were assessed by Cox regressions in a 1:1 propensity score-matched cohort. Of 8188 patients enrolled in 2017–21, 9% received a GLP-1 RA. Independent predictors of GLP-1 RA use were age <75 years, worse glycaemic control, impaired renal function, obesity, and reduced ejection fraction (EF). GLP-1 RA use was not significantly associated with a composite of HF hospitalization (HHF) or cardiovascular (CV) death regardless of EF, but was associated with a lower risk of major adverse CV events (CV death, non-fatal stroke/transient ischaemic attack, or myocardial infarction), and CV and all-cause death. In patients with body mass index ≥30 kg/m^2^, GLP-1 RA use was also associated with a lower risk of HHF/CV death and HHF alone.

**Conclusions:**

In patients with HF and T2DM, GLP-1 RA use was independently associated with more severe T2DM, reduced EF, and obesity and was not associated with a higher risk of HHF/CV death but with longer survival and less major CV adverse events. An association with lower HHF/CV death and HHF was observed in obese patients. Our findings provide new insights into GLP-1 RA use and its safety in HF and T2DM.

## Graphical Abstract

**Graphical Abstract:**
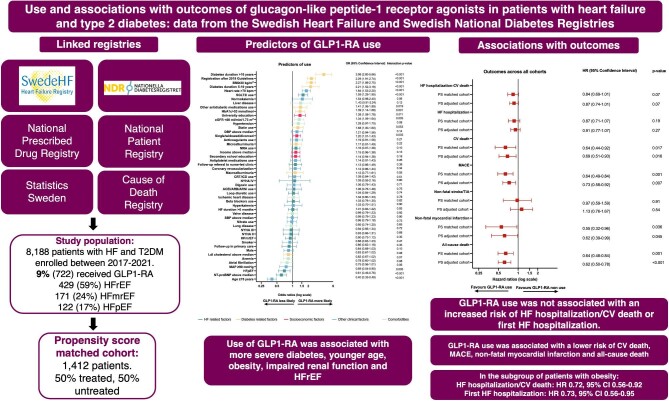

## Introduction

Heart failure (HF) and type 2 diabetes mellitus (T2DM) are two major public health problems, and patients with coexistent HF and T2DM have a poorer prognosis than those with only one of these two conditions.^1,2^

Glucagon-like peptide-1 receptor agonists (GLP-1 RA) are glucose-lowering drugs that reduce the risk of major adverse cardiovascular events (MACE) in patients with T2DM and high cardiovascular (CV) risk.^3^ This pharmacological class shows several effects that could potentially be favourable in HF, including weight loss, an increase in urinary sodium excretion, vasodilation, increases in the levels of endogenous natriuretic peptides, and the suppression of the renin–angiotensin system,^4,5^ but also induce an increase in heart rate and activate cyclic adenosine monophosphate-dependent pathways that might be prognostically unfavourable.^6^

In a meta-analysis of the FIGHT (Functional Impact of GLP-1 for Heart Failure Treatment) and the EXSCEL (Exenatide Study of Cardiovascular Event Lowering) trials, the use of the GLP-1 RA led to a higher risk of HF hospitalization in patients with HF and an ejection fraction (EF) <40%, whereas in a meta-analysis of RCTs (Randomised Controlled Trials) in patients with T2DM, the risk of HF hospitalization and mortality was not increased with GLP-1 RA.^7,8^ These signals of a potential detrimental effect of GLP-1 RA in patients with HF are worrisome, especially considering that liraglutide, semaglutide, and dulaglutide have Class IA recommendation in patients with T2DM and at high CV risk to reduce CV events according to international guidelines on diabetes.^9,10^ Additionally, GLP-1 RA could have a different prognostic role in patients with HF with preserved ejection fraction (HFpEF) vs. HF with reduced ejection fraction (HFrEF) due to the differences in pathophysiology in HF across the EF spectrum.^11^

The aims of the current study were to investigate GLP-1 RA use, patient characteristics associated with their use, and its associations with mortality/morbidity in an unselected cohort of HF patients with T2DM across the EF spectrum.

## Methods

### Data sources

The study population was derived from the Swedish Heart Failure Registry (SwedeHF), which was linked to the Swedish National Diabetes Registry, the National Patient Registry, the Cause of Death Registry, the Prescribed Drug Registry, and Statistics Sweden. Full description of the data sources is reported in the Supplemental Methods (see Supplementary material online, *Table S1*).

### Study population

Patients registered in SwedeHF between 1 January 2017 and 31 December 2021 were included (see Supplementary material online, *Table S2*). The index date was defined as the date of registration in SwedeHF, i.e. the date of the visit for outpatients and date of discharge for inpatients. The first registration was considered. A patient was defined as having T2DM if the patient (i) had been registered in the National Diabetes Registry prior to index date; (ii) was recorded as having T2DM at index date in SwedeHF; and (iii) had T2DM as comorbidity prior to index date according to the National Patient Registry.

### Statistical analysis

Categorical variables were reported as numbers (percentages) and compared using a χ^2^ test, whereas continuous variables were reported as medians (interquartile range—IQR) and compared by the Mann–Whitney test according to GLP-1 RA use.

Patients’ characteristics associated with GLP-1 RA use were investigated by univariable and multivariable logistic regression models, both in the overall population and according to EF by adding an interaction term between GLP-1 RA use and the EF class [HFpEF:EF ≥ 50%, HF with mildly reduced ejection fraction (HFmrEF):EF = 40–49%, HFrEF:EF < 40%]. To handle missing data for the variables included in the multivariable models, multiple imputation was performed (10 interactions; 10 databases generated); the variables included in the models are specified in *Table 1*.

**Table 1:** Baseline characteristics of patients receiving vs. not receiving a glucagon-like peptide-1 receptor agonist, for both the overall population and the matched population

	Overall cohort						Matched cohort			
	Untreated	Missing values (%)	Treated	Missing values (%)	*P*-value	ASD (%)	Untreated	Treated	*P*-value	ASD (%)
*n* (%)	7466 (91)		722 (9)				706 (50)	706 (50)		
**Demographics/organizational**										
Sex, female^a,b^	2199 (29)	0	199 (28)	0	0.29	4.2	188 (27)	192 (27)	0.81	1.3
Age ≥75 years^a,b^	4026 (54)	0	197 (27)	0	<0.001	5.6	186 (26)	197 (28)	0.51	3.5
Age, median (IQR)	75 (69–81)		70 (62–75)		<0.001	61.4	70 (64–75)	70 (62–75)	0.13	8.0
Follow-up referral to nurse-led clinic^a,b^	5847 (83)	5.6	610 (89)	5.0	<0.001	17.1	607 (89)	594 (89)	0.84	1.1
Centre of follow-up^a,b^		0		0	0.001	13.5			1.00	<0.001
** **Speciality care	6563 (88)		664 (92)				648 (92)	648 (92)		
Primary care	903 (12)		58 (8)				58 (8)	58 (8)		
**Clinical variables**										
Registration before 2019 guidelines^a,b^	4774 (64)	0	325 (45)	0	<0.001	38.7	326 (46)	325 (46)	0.96	0.3
EF^a,b^		0		0	<0.001	20.6			0.70	4.5
HFrEF	3829 (51)		429 (59)				430 (61)	417 (59)		
HFmrEF	1785 (24)		171 (24)				155 (22)	168 (24)		
HFpEF	1852 (25)		122 (17)				121 (17)	121 (17)		
Smoking^a,b^	529 (10)	28.1	51 (10)	28.3	1.00	0.0	57 (11)	49 (10)	0.66	2.7
HF <6 months^a,b^	3011 (42)	3.7	300 (43)		0.64	1.8	310 (45)	293 (43)	0.35	5.0
NYHA class^a,b^		23.6		24.4	0.61	6.0			0.29	11.6
I	458 (8)		49 (9)				39 (7)	48 (9)		
II	2680 (47)		241 (44)				288 (49)	235 (44)		
III	2461 (43)		245 (45)				251 (43)	238 (45)		
IV	106 (2)		11 (2)				11 (2)	11 (2)		
MAP <90 mm Hg^a,b^	3617 (50)	2.3	363 (52)	3.8	0.18	5.3	342 (50)	353 (52)	0.43	4.3
MAP, median (IQR)	91 (83–100)		90 (83–98)		0.45	2.3	90 (82–100)	90 (83–98)	0.71	−2.0
Heart rate ≤0 b.p.m.^a,b^	3434 (47)	3.1	222 (32)	3.5	<0.001	32.3	218 (32)	222 (33)	0.79	1.5
Anaemia^a,b^	2992 (40)	11.4	234 (32)	11.9	<0.001	17.3	199 (31)	202 (33)	0.53	3.5
Potassium^a,b^		3.4		3.2	0.62	3.7			0.45	6.9
Hypokalaemia	6613 (92)		634 (91)				615 (89)	618 (90)		
Normokalaemia	239 (3)		27 (4)				26 (4)	27 (4)		
Hyperkalaemia	357 (5)		39 (5)				50 (7)	38 (6)		
eGFR <60 mL/min/1.73 m^2^^a,b^	3238 (45)	2.7	299 (43)	3.2	0.36	3.6	290 (42)	293 (43)	0.66	2.4
NT-proBNP, above median^a,b^	4569 (61)	20.0	337 (47)	18.6	<0.001	29.4	200 (34)	202 (35)	0.72	2.1
BMI ≥30 kg/m^2^^a,b^	2742 (41)	35.9	438 (67)	33.0	<0.001	54.5	420 (66)	423 (66)	0.83	1.2
Atrial fibrillation^a,b^	4349 (58)	0	347 (48)	0	<0.001	20.5	353 (50)	343 (49)	0.59	2.8
Hypertension^a,b^	6435 (86)	0	648 (90)	0	0.008	11.0	627 (89)	632 (90)	0.67	2.3
Lung disease^a,b^	1204 (16)	2.2	109 (16)	2.8	0.51	2.6	107 (15)	109 (16)	0.84	1.1
Coronary revascularization^a,b^	2847 (39)	3.0	321 (46)	3.2	<0.001	13.4	304 (44)	313 (46)	0.54	3.3
Ischaemic heart disease^a,b^	4591 (61)	0	474 (66)	0	0.028	8.7	461 (65)	463 (66)	0.91	0.6
Valve disease^a,b^	1756 (24)	0	134 (19)	0	0.003	12.2	119 (17)	132 (19)	0.37	4.8
Liver disease^a,b^	204 (3)	0	27 (4)	0	0.12	5.7	26 (4)	25 (4)	0.89	0.8
Diabetes duration^a,b^		0		0	<0.001	32.0			0.18	9.9
<5 years	1043 (14)		47 (7)				61 (9)	47 (7)		
5–10 years	1852 (25)		134 (19)				113 (16)	133 (19)		
>10 years	4571 (61)		541 (75)				532 (75)	526 (75)		
HbA1c^a,b^ >53 mmol/mol	2702 (47)	23.0	385 (67)	20.8	<0.001	41.9	352 (66)	372 (67)	0.83	1.3
LDL-C, above median^a,b^	1907 (50)	49.2	158 (38)	42.8	<0.001	24.4	160 (45)	156 (39)	0.08	12.9
Albuminuria^a,b^		51.6		52.1	0.047	13.8			0.52	8.9
Normalized value	2211 (61)		188 (54)				193 (58)	185 (54)		
Microalbuminuria	1003 (28)		112 (32)				94 (28)	110 (32)		
Macroalbuminuria	403 (11)		46 (13)				44 (13)	46 (13)		
**Treatments**										
Loop diuretic^a,b^	5744 (77)	0.2	539 (75)	0.4	0.20	4.9	529 (75)	528 (75)	0.94	0.4
Statins^a,b^	5394 (72)	0.2	600 (83)	0.1	<0.001	26.2	592 (84)	585 (83)	0.62	2.7
Nitrates^a,b^	992 (13)	0	101 (14)	0	0.60	2.1	207 (29)	212 (30)	0.77	1.6
SGLT2i^a,b^	995 (13)	0	233 (32)	0	<0.001	46.4	223 (32)	219 (31)	0.82	1.7
ACEi/ARB/ARNI^a,b^	6733 (90)	0	672 (93)	0	0.012	10.5	659 (93)	656 (93)	0.75	1.8
Beta-blockers^a,b^	6493 (87)	0	642 (89)	0	0.13	6.0	622 (88)	626 (89)	0.74	1.8
MRA^a,b^	3493 (47)	0	391 (54)	0	<0.001	14.8	376 (53)	381 (54)	0.79	1.4
Other antidiabetic medications^a,b^	5875 (79)	0	657 (91)	0	<0.001	34.8	635 (90)	641 (91)	0.59	2.9
Digoxin^a,b^	826 (11)	0	70 (10)	0	0.26	4.5	80 (11)	69 (10)	0.34	5.1
Anticoagulants^a,b^	4214 (56)	0	369 (51)	0	0.006	10.7	374 (53)	362 (51)	0.52	3.4
Antiplatelet medications^a,b^	3293 (44)	0	383 (53)	0	<0.001	18.0	356 (50)	371 (53)	0.42	4.3
CRT/ICD^a,b^	843 (11)	0.7	106 (15)	0.1	0.008	9.9	98 (14)	103 (15)	0.71	2.0
**Socioeconomic variables**										
Marital status^a,b^		0.1		0	0.06	7.2			0.59	2.8
Married	3730 (50)		335 (46)				321 (45)	331 (47)		
Single/widowed/divorced	3728 (50)		387 (54)				385 (55)	375 (53)		
Education^a,b^		1.7		1.1	<0.001	16.3			0.65	4.9
Compulsory school	3009 (41)		240 (34)				246 (35)	236 (34)		
Secondary school	3177 (43)		333 (47)				325 (47)	323 (46)		
University	1150 (16)		141 (20)				126 (18)	139 (20)		
Income, below median^a,b^	3778 (51)	0.1	314 (43)	0	<0.001	14.4	326 (46)	310 (44)	0.39	4.6

ACEi, angiotensin-converting enzyme inhibitors; ARB, angiotensin receptor blockers; ARNI, angiotensin receptor–neprilysin inhibitors; ASD, absolute standardized difference; BMI, body mass index; b.p.m., beats per minute; CRT, cardiac resynchronization therapy; DBP, diastolic blood pressure; EF, ejection fraction; eGFR, estimated glomerular filtration rate; HbA1c, glycated haemoglobin; HF, heart failure; HFmrEF, heart failure with mildly reduced ejection fraction; HFpEF, heart failure with preserved ejection fraction; HFrEF, heart failure with reduced ejection fraction; ICD, implantable cardioverter defibrillator; IQR, interquartile range; LDL-C, low-density lipoprotein cholesterol; MAP, mean arterial pressure; MRA, mineralocorticoid receptor antagonists; NT-proBNP, N-terminal pro-B-type natriuretic peptide; NYHA, New York Heart Association; SBP, systolic blood pressure; SGLT2i, sodium-glucose cotransporter 2 inhibitors.

a Variables used for multiple imputation.

b Variables used to estimate propensity score and GLP-1 RA use.

The primary outcome was time to a composite of HF hospitalization or CV death. Secondary outcomes were time to HF hospitalization, CV death, a composite of major adverse CV events [MACE, i.e. CV death, non-fatal stroke/transient ischaemic attack (TIA), and non-fatal myocardial infarction], non-fatal stroke/TIA, non-fatal myocardial infarction, all-cause death, and repeated HF hospitalizations.

Propensity scores (PS) for the use of GLP-1 RA were calculated within each imputed dataset using a logistic regression model including the variables indicated in *Table 1* and then averaged across the 10 imputed datasets. Matching was performed 1:1 by the nearest neighbour method without replacement and a calliper ≤0.01. Matching balance for patients’ baseline characteristics was deemed appropriate if the absolute standardized differences were ≤10%.

To investigate the association between GLP-1 RA use and outcomes, univariable Cox proportional hazards regression models were fitted (i) in the overall population (unadjusted results) and (ii) in the PS-matched population (accounting for within matched-pairs dependence) to provide adjusted results. Due to the expected reduction in sample size with PS matching, we also performed analyses adjusting rather than matching for the PS in the overall cohort. Subgroup analyses were performed in the PS-matched cohort by including an interaction term between selected variables and GLP-1 RA use in the Cox regression models. Separate outcome analyses were performed in the subgroup of patients with obesity and, also according to EF, in the subgroups of patients with age <75 or ≥75 years (median value) and in the subgroups of patients with a body mass index (BMI) ≥25 kg/m^2^ only. The proportionality of hazards was tested by Schoenfeld residuals. The association between GLP-1 RA use and repeated HF hospitalizations was investigated by a negative binomial regression, and the results were expressed as incidence rate ratios (IRR) with 95% confidence intervals (CI).

All analyses were performed using Stata version 16.1 (Stata Corp., College Station, TX). A *P*-value <0.05 was considered as statistically significant.

## Results

Between 1 January 2017 and 31 December 2021, there were 8188 patients with both HF and T2DM registered in SwedeHF and fulfilling the selection criteria for the current study. Median age was 75 years (IQR = 68–80), 29% were female, and 52%, 24%, and 24% with HFrEF, HFmrEF, and HFpEF, respectively.

In total, 722 patients (9%) were treated with a GLP-1 RA, and more specifically 6% in HFpEF, 9% in HFmrEF, and 10% in HFrEF. Within the GLP-1 RA-treated group, the most prescribed drug was liraglutide (59%), followed by semaglutide (24%), dulaglutide (13%), and exenatide or lixisenatide (4%). The number of patients initiated with a GLP-1 RA increased gradually over time, i.e. 116 (5%) in 2017 to 196 (16%) in 2021 (see Supplementary material online, *Figure S1*).

### Patient characteristics according to GLP-1 RA use

Patients treated with a GLP-1 RA were younger, more likely obese and with HFrEF, had significantly lower levels of N-terminal pro-B-type natriuretic peptide (NT-proBNP), with a history of ischaemic heart disease, renal impairment, a longer duration of T2DM and a worse glycaemic control (i.e. higher prevalence of retinopathy and albuminuria), and higher education level and income compared with patients not on GLP-1 RA (*Table 1*). GLP-1 RA users were more likely to receive medical therapy for HF [mineralocorticoid receptor antagonists, sodium-glucose cotransporter 2 inhibitors (SGLT2i), angiotensin-converting enzyme inhibitors/angiotensin receptor blockers/angiotensin receptor–neprilysin inhibitor, and HF devices], and followed up in nurse-led clinics and speciality vs. primary care. Use of SGLT2i was more common in GLP-1 RA user vs. non-users (32% vs. 13%, *P* < 0.001), as well as that of other antidiabetic medications (91% vs. 79%, *P* < 0.001).

### Independent predictors of GLP-1 RA use

**Figure 1:**
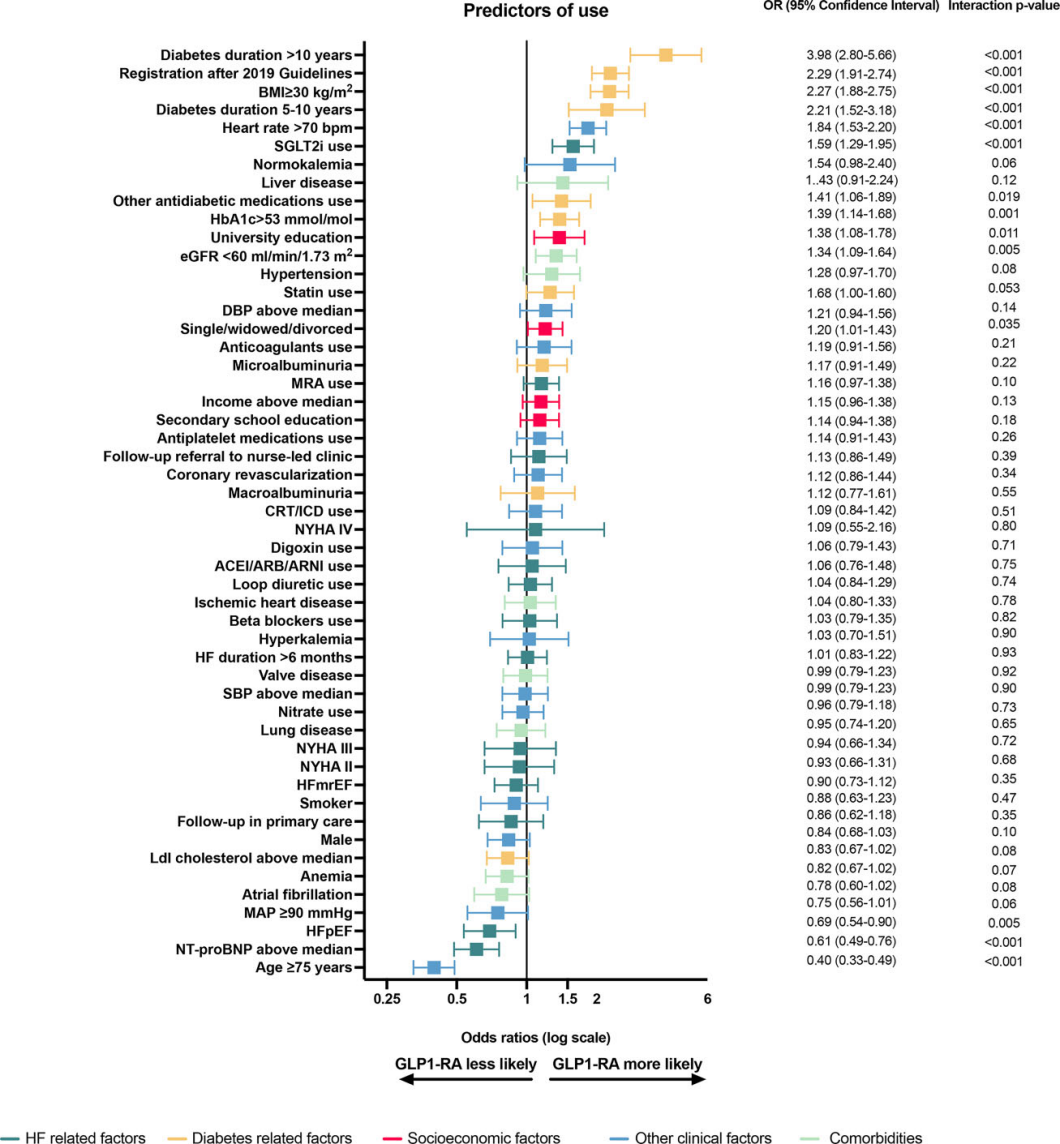
Independent predictors of GLP-1 RA use. Abbreviations as in *Table 1*.

Independent predictors associated with GLP-1 RA use were age <75 years, having HFrEF and a longer duration of T2DM, obesity, registration in SwedeHF after release of the 2019 European Society of Cardiology/European Association for the Study of Diabetes (ESC/EASD) guidelines, heart rate >70 b.p.m., glycated haemoglobin A1c (HbA1c) >53 mmol/mol, lower low-density lipoprotein cholesterol levels and NT-proBNP, university education, concomitant use of SGLT2i or other antidiabetic medications, and an estimated glomerular filtration rate (eGFR) <60 mL/min/1.73 m² (*Figure 1*).

Few predictors of GLP-1 RA use differed across the EF subtypes (see *S4*). The magnitude of the association between higher heart rate (>70 b.p.m.) and GLP-1 RA use was greater in HFrEF vs. HFmrEF, with the association not being statistically significant in HFpEF (*P*-value for interaction: 0.019); anticoagulant use was associated with a higher use of GLP-1 RA only in HFpEF (*P*-value for interaction: 0.035); registration after the release of the 2019 guidelines was associated with a higher use of GLP-1 RA in all HF classes, although significantly more in HFmrEF and HFpEF than in HFrEF (*P*-value for interaction <0.001).

### Outcome analyses

**Figure 2:**
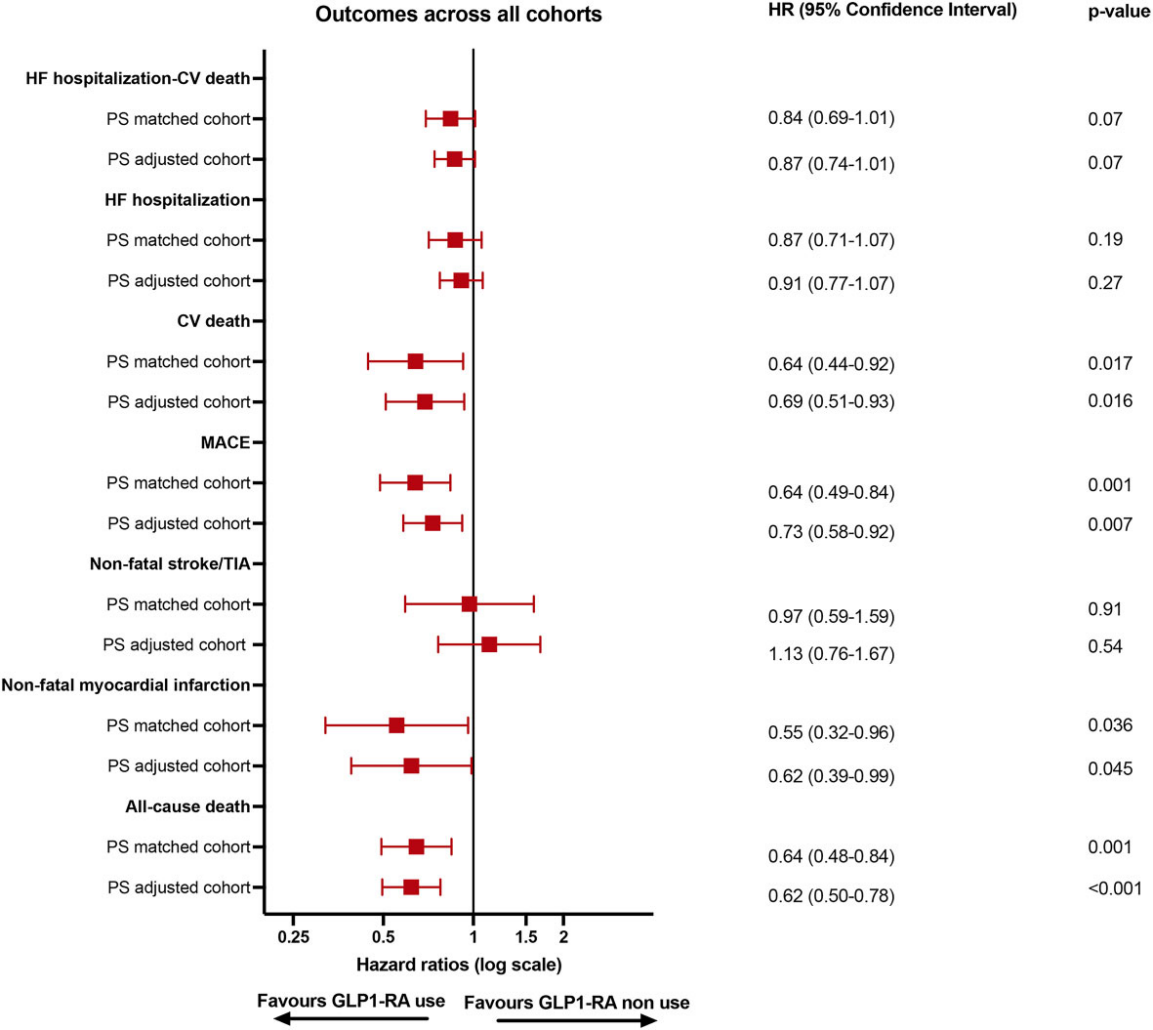
Outcome analysis. PS, propensity score; HR, hazard ratio; CI, confidence interval; HF, heart failure; CV, cardiovascular; MACE, major adverse cardiovascular events; TIA, transient ischaemic attack; GLP-1 RA, glucagon-like peptide-1 receptor agonists.

Over a median follow-up time of 1.6 years (IQR = 0.6–2.9), event rates for the primary outcome (HF hospitalization or CV death) in the overall cohort for patients receiving vs. not receiving GLP-1 RA were 15.7 vs. 19.4/100 patient-years, respectively (*Figure 2*, see Supplementary material online, *Table S3* and *Figure S5*). Corresponding event rates in the PS-matched population were 15.8 and 19.5/100 patient-years, which translated into an HR of 0.84 (95% CI: 0.69–1.01).

As regards secondary outcomes, the HR for the association of GLP-1 RA use with a first HF hospitalization in the PS-matched cohort was 0.87 (95% CI: 0.71–1.07); GLP-1 RA use was associated both with a 36% lower risk of CV death (HR: 0.64, 95% CI: 0.44–0.92), MACE (HR: 0.64, 95% CI: 0.49–0.84), and all-cause death (HR: 0.64, 95% CI: 0.48–0.84) and with a 45% lower risk of non-fatal myocardial infarction (HR: 0.55, 95% CI: 0.32–0.96), whereas there was no statistically significant association with the risk of non-fatal stroke/TIA (HR: 0.97, 95% CI: 0.59–1.59) and repeated HF hospitalizations (IRR: 0.80, 95% CI: 0.58–1.11). These results were consistent in PS-adjusted analysis.

Kaplan–Meier curves for outcomes in the propensity score-matched cohort are reported in Supplementary material online, *Figure S5*.

### Subgroup analysis

**Figure 3:**
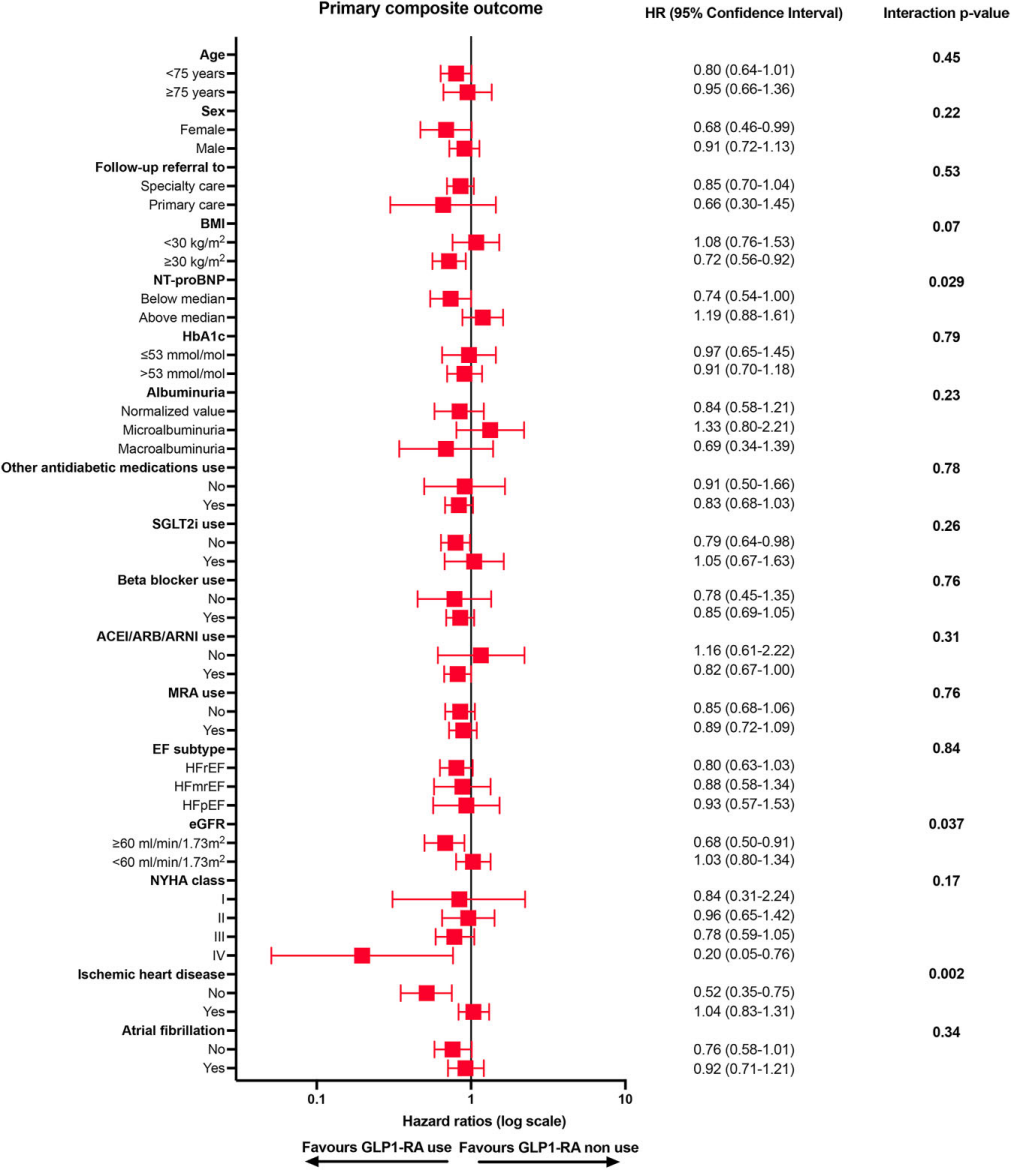
Subgroup analysis for the primary composite outcome performed in the propensity score-matched cohort. Abbreviations as in *Table 1*.

The association between GLP-1 RA use and the primary composite endpoint was generally consistent across several subgroups in the PS-matched cohort, except for an associated lower risk in patients without ischaemic heart disease but not in those with ischaemic heart disease (*P*-value for interaction: 0.002), and in patients with preserved renal function vs. those with impaired renal function (*P*-value for interaction: 0.037) (*Figure 3*, see Supplementary material online, *Table S4*–*S7* and *Figure S6*).

The associations between GLP-1 RA use and outcomes were also separately analysed in HFrEF, HFmrEF, and HFpEF as reported in Supplementary material online, *Table S4* and *Figure S6*. Overall results were consistent across the EF subtypes.

We conducted the outcome analysis, both in the PS-matched population and in the PS-adjusted population for consistency, separately in patients with a BMI ≥25 and ≥30 kg/m^2^. In the subgroup of patients with BMI ≥25 kg/m^2^, the associations with all outcomes were consistent with the results in the overall population (see Supplementary material online, *Table S5*). In those with a BMI ≥30 kg/m^2^, GLP-1 RA use was associated with a statistically significant lower risk of the primary composite outcome (HR: 0.72, 95% CI: 0.56–0.92) and first HF hospitalization (HR: 0.73, 95% CI: 0.56–0.95), and all the other outcomes except stroke/TIA and repeated HF hospitalizations. All results were consistent across the EF strata, and in the PS-adjusted analysis except for the association of GLP-1 RA use with a significant lower risk of HF hospitalization (IRR: 0.76, 95% CI: 0.59–0.98; see Supplementary material online, *Table S6*).

The associations between GLP-1 RA use and outcomes were consistent regardless of age category (see Supplementary material online, *Table S7*).

## Discussion

In this nationwide, real-world cohort of patients with HF and T2DM, we observed that (i) the use of GLP-1 RA increased over time, up to 16% in 2021; (ii) the main patient characteristics independently associated with GLP-1 RA use were younger age, long-standing T2DM with poor glycaemic control, impaired renal function, obesity, and having HFrEF; and (iii) the use of GLP-1 RA was not associated with a higher risk of CV death/HF hospitalization or HF hospitalization alone, neither as first event nor as repeated event, and was associated with a lower risk of MACE, myocardial infarction, and mortality. These results were overall consistent across the EF spectrum. Although there was no formal statistically significant interaction for the association between GLP-1 RA use and the primary outcome in patients with vs. without obesity (*P*-value for interaction: 0.07), in the stratum of patients with a BMI ≥30 kg/m^2^, use of GLP-1 RA was associated with a statistically significant lower risk of CV death or HF hospitalization, as well as HF hospitalization, CV and all-cause death, and MACE regardless of EF.

### Use and independent predictors of use of GLP-1 RA

To date, several GLP-1 RA have been tested in CV outcome trials (CVOTs) in patients with T2DM and high CV risk, with liraglutide, semaglutide, dulaglutide, albiglutide, and efpeglenatide being superior to placebo in reducing the incidence of MACE, while lixisenatide and exenatide did not achieve superiority.^12^ Our results show a gradual increase in the prescription of GLP-1 RA, from 5% in 2017 up to 16% in 2021. The increase was greater after 2019, when the previous European guidelines on diabetes and CV disease were released, with an index date after 2019 being a significant predictor of use in our analysis.

Younger age was an independent predictor of treatment, as previously reported for renin–angiotensin–aldosterone inhibitors and SGLT2i use, and might be explained by the attempt of minimizing tolerability issues and adverse effects that might be more likely in older patients. Potential beneficial effects in older and frailer patients tend to be underestimated due to comorbidities, competing risk, and lower representation in randomized trials: the mean age of patients enrolled in GLP-1 RA CVOTs ranged 60–66 years.^13,14^ The association with long-standing T2DM, poor glycaemic control, and the use of other glucose-lowering drugs might reflect GLP-1 RA not being considered yet first-line treatments for T2DM, and consistently they are still recommended after metformin according to Swedish local guidelines. Impaired renal function was also among the independent predictors of use, and indeed GLP-1 RA can be used in chronic kidney disease with an eGFR ≥15 mL/min/1.73 m², while metformin is contraindicated with an eGFR <30 mL/min/1.73 m². GLP-1 RA have demonstrated a sustained weight reduction in CVOTs and are recommended in patients with T2DM and obesity.^15^ It is therefore not surprising that in our analysis a BMI ≥30 kg/m² was associated with higher likelihood of use. HFrEF was independently associated with more frequent use of GLP-1 RA compared with HFpEF, which possibly linked with the perception of the need of a more intensive treatment in patients with HFrEF since they are at higher risk of outcomes. However, predictors of GLP-1 RA did not substantially differ across the EF spectrum. Finally, the associations with lower NT-proBNP levels and a higher heart rate could reflect biological effects of GLP-1 RA.^6,16^ The effect on heart rate should not discourage from the use of GLP-1 RA in HF; instead, it needs to be counteracted with appropriate re-evaluation and dose optimization of beta-blockers and ivabradine.

### Associations between GLP-1 RA use and outcomes

The safety of glucose-lowering drugs in HF has been much debated, since an increased risk of incident HF was reported with other classes of glucose-lowering drugs, e.g. thiazolidinediones and saxagliptin. Generally, GLP-1 RA trials were underpowered to detect either an effect in HF patients, with HF prevalence in trial populations only being 9–24%, or a risk reduction of HF events.^3^ A meta-analysis of pooled data from all GLP-1 RA CVOTs in T2DM up to 2019 reported a statistically significant 9% reduction in risk of HF hospitalization, possibly mediated by GLP-1 RA positive effects on CV risk factors.^3,4^ When assessing the effect of GLP-1 RA separately in patients with and without HF, a benefit was reported in patients without but not in those with a history of HF.^8^ Liraglutide did neither improve clinical stability after a hospitalization for HF in the FIGHT trial nor increase EF in the LIVE trial.^17,18^ On the contrary, a *post hoc* analysis of the FIGHT trial reported a trend towards an increased risk of HF hospitalization and mortality events with liraglutide in patients with HFrEF, consistent with findings in the HFrEF subgroup of the EXSCEL trial having a significantly higher risk of HF hospitalization with exenatide.^7,19^ Consistently, in a pooled analysis of Trial to Evaluate Cardiovascular and Other Long-term Outcomes with Semaglutide in Subjects with Type 2 Diabetes (SUSTAIN-6) and Peptide Innovation for Early Diabetes Treatment (PIONEER)-6, semaglutide reduced the risk of the composite of CV death, myocardial infarction, or stroke in all subgroups, except for those with an HF history.^20^

We did not find any association between GLP-1 RA use and a higher risk of HF hospitalization or CV death, and rather the trend was towards a lower risk (*P*-value: 0.07), mainly driven by a statistically significant association with a 36% lower risk of CV death. There was also a statistically significant association between GLP-1 RA use and a lower risk of MACE, non-fatal myocardial infarction, and all-cause death, consistently with CVOTs, but we reported higher event rates as expected in a real-world population.^14^ Interestingly, we found an interaction between ischaemic heart disease and GLP-1 RA use for the association with CV mortality or HF hospitalization, with a lower risk in those receiving the treatment if they did not have a history of ischemic heart disease. We speculate that this finding, in the context of our overall results, might suggest a role for GLP-1 RA in HF that is not mediated by an effect on atherosclerotic events and/or that the better outcome with GLP-1 RA in non-ischaemic HF might be more likely mediated by weight loss. The association of GLP-1 RA use with a lower risk for the primary outcome in the subset with impaired renal function might reflect their benefit when other glucose-lowering drugs cannot be used or uptitrated.

Our results were consistent across the EF spectrum. To date, there is no RCT conducted in patients with HF across the EF spectrum investigating the effect of GLP-1 RA on these hard outcomes. In two RCTs in HFrEF, neither albiglutide nor liraglutide improved EF, myocardial function, or exercise capacity compared with placebo.^21,22^

We performed a separate outcome analysis in patients with obesity, even though the interaction term between GLP-1 RA use and the presence of obesity fell short of statistical significance by a small amount (*P*-value for interaction: 0.07), as the Semaglutide Treatment Effect in People with Obesity (STEP) programme trials are focusing on this patient subpopulation and showed that GLP-1 RA induce substantial weight loss in patients with overweight and obesity, both with and without T2DM,^23,24^ and in the Semaglutide for Cardiovascular Event Reduction in People with Overweight or Obesity (SELECT) trial patients with CV disease and overweight or obesity, but without diabetes subcutaneous semaglutide was superior to placebo in reducing MACE.^25^ We found that, in the subgroup with obesity, the use of GLP-1 RA was also associated with a significant 28% lower risk of the primary outcome and a 27% lower risk of HF hospitalization, with consistent results across the EF spectrum. Recently, the Semaglutide Treatment Effect in People with Obesity and HFpEF (STEP-HFpEF) and Semaglutide Treatment Effect in People with Obesity and HFpEF and Type 2 Diabetes (STEP-HFpEF DM) trials demonstrated that semaglutide improved symptoms and physical limitations, and exercise function, and induced weight loss in HFpEF without and with T2DM, respectively.^26,27^ We might speculate that our results could suggest a benefit on hard outcomes in patients with obesity and potentially extend the benefit found in HFpEF to the whole EF spectrum.

### Strengths and limitations

The linkage of several national registries allowed us to perform extensive adjustments; however, this was an observational study and residual confounding cannot be ruled out. In addition, our study is limited by the relatively short average follow-up. While the coverage of the National Diabetes Registry is almost 100%, SwedeHF only includes approximately one-third of HF patients in Sweden, which might be linked with selection bias. Finally, our findings are representative of Sweden but might be limitedly generalizable to other countries.

## Conclusions

In patients with HF and T2DM, the use of GLP-1 RA was independently associated with HFrEF and more severe T2DM. We found no association between GLP-1 RA use and a higher risk of the composite of HF hospitalization or CV death, or HF hospitalizations, which reassures on the safety of these drugs in the setting of T2DM with concomitant HF. Our finding of a lower risk of CV death or HF hospitalization and of a lower risk of HF hospitalization in patients with obesity might suggest a role of GLP-1 RA on hard outcomes in patients with obesity and HF across the EF.

## Supplementary Material

pvae026_Supplemental_File

## Notes

**Conflict of interest:** M.W. reports personal fees from Vifor CSL, Boehringer Ingelheim, Novartis, Bayer, and AstraZeneca, outside of the submitted work. M.E.B. has no conflicts of interest related to this work. D.S. reports personal fees from AstraZeneca, Novartis, Novo Nordisk, Merck, and Janssen, outside of the submitted work. G.S. reports personal fees for educational activities (Biotronik, Boston Scientific, AstraZeneca, Novartis, Menarini, and Vifor Pharma) outside of the submitted work. L.B. has no conflicts of interest related to this work. U.D. reports grants from Pfizer, Vifor, Boehringer Ingelheim, AstraZeneca, Boston Scientific, and Roche Diagnostics and honoraria/consultancies from Amgen and AstraZeneca, all outside of the submitted work. S.G. has no conflicts of interest related to this work. F.C. has no conflicts of interest related to this work. P.G.M.M. has no conflicts of interest related to this work. G.M.C.R. has no conflicts of interest related to this work. J.B. reports consultant fees from Abbott, American Regent, Amgen, Applied Therapeutic, AstraZeneca, Bayer, Boehringer Ingelheim, Bristol Myers Squibb, Cardiac Dimension, Cardior, CVRx, Cytokinetics, Edwards, Element Science, Innolife, Impulse Dynamics, Imbria, Inventiva, Lexicon, Lilly, LivaNova, Janssen, Medtronics, Merck, Occlutech, Novartis, Novo Nordisk, Pfizer, Pharmacosmos, Pharmain, Roche, Sequana, SQ Innovation, and Vifor, outside of the submitted work. M.M. has no conflicts of interest related to this work. L.L. reports grants, consulting, and honoraria from Myocardia, AstraZeneca, Boehringer Ingelheim/Lilly, Novartis, Bayer, Vifor, Sanofi/Lexicon, Servier, Abbott, Pharmacosmos, Medscape, Radcliffe, TMA, Orion Pharma, and Alleviant, outside of the submitted work, and ownership of AnaCardio. G.F. reports personal fees from Vifor, AstraZeneca and Novartis, outside of the submitted work. G.S. reports grants and personal fees from Vifor, grants and personal fees from Boehringer Ingelheim, grants and personal fees from AstraZeneca, personal fees from Servier, grants and personal fees from Novartis, grants and personal fees from Cytokinetics, personal fees from Medtronic, grants from Boston Scientific, grants and personal fees from Pharmacosmos, grants from Merck, grants from Bayer, personal fees from TEVA, personal fees from INTAS, personal fees from Abbott, and personal fees from Edwards Lifescience, outside of the submitted work.

## Data availability

The data underlying this article will be shared on reasonable request to the corresponding author.
