# Supplementary material for: Glucagon-like peptide-1 receptor agonists use and associations with outcomes in heart failure and type 2 diabetes: data from the Swedish Heart Failure and Swedish National Diabetes Registries
Source: Eur Heart J Cardiovasc Pharmacother. 2024 Apr 17;10(4):296–306. doi: 10.1093/ehjcvp/pvae026 (PMC11250228; doi:10.1093/ehjcvp/pvae026)
Supplement: pvae026_Supplemental_File [file pvae026_supplemental_file.docx]

**Supplemental material**

**Methods**

SwedeHF and the National Diabetes Registry are quality and research registries, whose designs have previously been described^1^. Briefly, between 2000 and 2017 SwedeHF has enrolled patients with a diagnosis of clinician-judged HF and, thereafter, HF has been defined according to the International Statistical Classification of Diseases, Tenth Revision (ICD-10) codes I50.0, I50.1, I50.9, I42.0, I42.6, I42.7, I25.5, I11.0, I13.0, I13.2. Approximately 80 variables (i.e. demographics, comorbidities, clinical variables, laboratory measurements and organizational information) are recorded at hospital discharge or at an outpatient visit, i.e. index date.^1^ At the end of 2021, the coverage of SwedeHF was 32% of the prevalent HF population in Sweden.^2^ The National Diabetes Registry was started in 1996 and has enrolled patients with diabetes encountered in outpatient clinics and primary care. Data on demographics, risk factors, complications of diabetes and medications are collected. In 2021, the coverage of National Diabetes Registry for T2DM was 85%.^3^ Linkage with the National Patient Registry provided additional data on comorbidities and cause-specific hospitalization as outcome, while linkage with the Cause of Death Registry provided information on the date of death and cause-specific mortality. Definitions of variables are reported in **Supplemental Table 1**. Statistics Sweden provided data on the patients´ socioeconomic status, whereas linkage with the Prescribed Drug Registry provided additional data about medications, and in particular regarding the exposure of the current study, i.e. GLP-1 RA use, which was defined as a dispensed prescription five months prior to 14 days after the index date. The data sources for the individual variables included in this analysis are reported in **Supplemental Table 1**. These registries were linked by the Swedish personal identification number, which every resident in Sweden has.

Individual consent to enrolment in SwedeHF is not necessary, but patients are allowed to opt out. In NDR, each patient provides oral or written informed consent for inclusion in the register. The linkage of the registries for this study has been approved by the Swedish Ethical Review Authority in Stockholm, with Dnr 2021-04326.

**Supplemental Table 1**. Variable sources.

| **Variables** | **Data source** |
| --- | --- |
| Index date | SwedeHF |
| EF | SwedeHF |
| Gender | SwedeHF |
| Age | SwedeHF |
| Smoke | SwedeHF |
| HF duration | SwedeHF |
| NYHA class | SwedeHF |
| SBP | SwedeHF |
| DBP | SwedeHF |
| Heart rate | SwedeHF |
| Anemia | SwedeHF |
| Potassium | SwedeHF |
| eGFR | SwedeHF |
| NT-proBNP | SwedeHF |
| Loop diuretics | SwedeHF |
| Statins | SwedeHF |
| Nitrates | PDR (ATC codes: C01DA) |
| Devices | SwedeHF |
| BMI | SwedeHF |
| Atrial fibrillation | NPR, SwedeHF (ICD-10 code: I48) |
| Hypertension | NPR, SwedeHF (ICD-10 codes: I10-5) |
| Lung disease | SwedeHF |
| Coronary revascularization | SwedeHF |
| Follow-up in nurse-led clinic | SwedeHF |
| Marital status | Statistics Sweden |
| Education | Statistics Sweden |
| Income | Statistics Sweden |
| Ischemic heart disease | NPR, SwedeHF (ICD-9 codes: I410-4. ICD-10 codes: I20-5) |
| SGLT2i | PDR (ATC codes: A10BK; A10BD15; A10BD16; A10BD19; A10BD20; A10BD21; A10BD23; A10BD24; A10BD25; A10BX09; A10BX11; A10BX12) |
| GLP1-RA | PDR (ATC codes: A10BJ; A10BX04; A10BX07; A10BX10; A10BX13; A10BX14) |
| ACEI/ARB/ARNI | PDR (ATC codes: C09A; C09B; C09C; C09D) |
| Beta-blockers | PDR (ATC codes: C07) |
| MRA | PDR (ATC codes: C03DA) |
| Diabetes duration | NDR |
| HbA1c | NDR |
| LDL cholesterol | NDR |
| Center of follow-up | SwedeHF |
| Metformin | PDR (ATC codes: A10BA02; A10BD02; A10BD03; A10BD05; A10BD07; A10BD08; A10BD10; A10BD11; A10BD13; A10BD14; A10BD15; A10BD16; A10BD17; A10BD18; A10BD20; A10BD22; A10BD23; A10BD25) |
| Insulin | PDR (ATC codes: A10A) |
| Glitazones | PDR (ATC codes: A10BG) |
| DPP4i | PDR (ATC codes: A10BH; A10BD07; A10BD08; A10BD09; A10BD10; A10BD11; A10BD12; A10BD13; A10BD18; A10BD19; A10BD21; A10BD22; A10BD24; A10BD25) |
| Digoxin | PDR (ATC code: C01AA05) |
| Anticoagulants | PDR (ATC codes: B01A excluding B01AC) |
| Antiplatelet medications | PDR (ATC codes: B01AC) |
| Valve disease | NPR, SwedeHF (ICD-10 codes: I05-8; I34-9; Q22; Q230-3; Q235-9; Z952-4) |
| Liver disease | NPR (ICD-10 codes: B18; I85; I864; I982; K70; K710; K711; K713-7; K72-4; K760; K762-9) |
| Albuminuria | NDR |
| Triglycerides | NDR |
| Diabetic retinopathy | NDR |
| Hospitalization for HF | NPR; (ICD-10 codes: I110; I130; I132; I255; I420; I423; I425; I426; I427; I428; I429; I43; I50; J81; K761; R570; 414W; 425E; 425F; 425G; 425H; 425W; 425X; 428. ICD-9 code: 428) |
| Count of HF hospitalizations | NPR; (ICD-10 codes: I110; I130; I132; I255; I420; I423; I425; I426; I427; I428; I429; I43; I50; J81; K761; R570; 414W; 425E; 425F; 425G; 425H; 425W; 425X; 428. ICD-9 code: 428) |
| CV death | Cause of death registry (ICD-10 codes: J81; K761; R570; G45) |
| Hospitalization for myocardial infarction | NPR (ICD-10 codes: I21; I22) |
| Hospitalization for stroke/TIA | NPR (ICD-10 codes: I60-4; G45) |
| All-cause death | Cause of death registry |

**List of abbreviations:** NDR, National Diabetes Register; NPR, National patient Register; PDR, Prescribed Drugs Register; LISA, Longitudinal Integration Database for Health Insurance and Labor Market Studies; ICD, International Classification of Diseases; ATC, Anatomical Therapeutic Chemical; ACE, Angiotensin Converting Enzyme; ARB, Angiotensin Receptor Blocker; ARNI, Angiotensin Receptor Neprilysin Inhibitor; BMI, Body Mass Index; CV, Cardiovascular; DBP, Diastolic Blood Pressure; DPP4i, Dipeptidyl Peptidase 4 inhibitor; EF, Ejection Fraction; GLP1-RA, Glucagon-like Peptide 1 Receptor Agonists; HbA1c, glycated hemoglobin; HF, Heart Failure; MRA, Mineralocorticoid Receptor Antagonists; NT-proBNP, N-terminal B-type Natriuretic peptide; NYHA, New York Heart Association; SBP, Systolic Blood Pressure; SGLT2i, Sodium-Glucose Cotransporter 2 inhibitors; TIA, Transient Ischemic Attack.

**Supplemental Table 2**. Patient selection.

| **Criteria** | **Number** |
| --- | --- |
| Registrations in SwedeHF database version 4 | 203428 |
| Index date after 1^st^ January 2017 | 75843 |
| Non-missing information on ejection fraction | 56203 |
| More than 14 days follow-up (to avoid immortal time bias) | 55608 |
| Select first registration in SwedeHF | 41613 |
| Exclude patients with diabetes type 2 and no registration in the National Diabetes Register within six months from index date | 37903 |
| Select patients with type 2 diabetes only | 8188 |

**Supplemental Table 3**. Number of events and hazard rations for the explored outcomes in the crude analysis, in the propensity-score matched population and in the analysis adjusted for the propensity score.

| **Outcome** | **Number of events**  **(per 100 person-years)** | | **Hazard ratio** | **95% Confidence Interval** | **p-value** |
| --- | --- | --- | --- | --- | --- |
|  | **Treated** | **Untreated** |  |  |  |
| **Primary composite** |  |  |  |  |  |
| Crude | 15.7 | 19.4 | 0.79 | 0.68-0.91 | 0.001 |
| PS matched | 15.8 | 19.0 | 0.84 | 0.69-1.01 | 0.07 |
| PS adjusted | - | - | 0.87 | 0.74-1.01 | 0.07 |
| **First HF hospitalization** |  |  |  |  |  |
| Crude | 14.1 | 16 | 0.85 | 0.73-1.00 | 0.045 |
| PS matched | 14.2 | 16.4 | 0.87 | 0.71-1.07 | 0.19 |
| PS adjusted | - | - | 0.91 | 0.77-1.07 | 0.27 |
| **CV death** |  |  |  |  |  |
| Crude | 3.2 | 6.4 | 0.49 | 0.37-0.66 | <0.001 |
| PS matched | 3.2 | 5.0 | 0.64 | 0.44-0.92 | 0.017 |
| PS adjusted | - | - | 0.69 | 0.51-0.93 | 0.016 |
| **MACE** |  |  |  |  |  |
| Crude | 6.0 | 9.9 | 0.60 | 0.48-0.75 | <0.001 |
| PS matched | 6.0 | 9.4 | 0.64 | 0.49-0.84 | 0.001 |
| PS adjusted | - | - | 0.73 | 0.58-0.92 | 0.007 |
| **Non-fatal stroke/TIA** |  |  |  |  |  |
| Crude | 2.1 | 2.3 | 0.90 | 0.62-1.31 | 0.58 |
| PS matched | 2.1 | 2.2 | 0.97 | 0.59-1.59 | 0.91 |
| PS adjusted | - | - | 1.13 | 0.76-1.67 | 0.54 |
| **Non-fatal myocardial infarction** |  |  |  |  |  |
| Crude | 1.4 | 2.1 | 0.68 | 0.44-1.06 | 0.09 |
| PS matched | 1.4 | 2.5 | 0.55 | 0.32-0.96 | 0.036 |
| PS adjusted | - | - | 0.62 | 0.39-0.99 | 0.045 |
| **All-cause death** |  |  |  |  |  |
| Crude | 5.7 | 12.7 | 0.45 | 0.36-0.56 | <0.001 |
| PS matched | 5.8 | 9.1 | 0.64 | 0.48-0.84 | 0.001 |
| PS adjusted | - | - | 0.62 | 0.50-0.78 | <0.001 |
| **Repeated HF hospitalizations*** |  |  |  |  |  |
| Crude | - | - | 0.77 | 0.73-0.81 | <0.001 |
| PS matched | - | - | 0.80 | 0.58-1.11 | 0.18 |
| PS adjusted | - | - | 0.83 | 0.68-1.03 | 0.09 |

* for repeated HF hospitalization, the point estimate is the incidence rate ratio.

**Legend**: PS, Propensity Score; HR, Hazard Ratio; CI, Confidence Interval; HF, Heart Failure; CV, Cardiovascular; MACE, Major Adverse Cardiovascular Events; TIA, Transient Ischemic Attack.

**Supplemental Table 4**. Number of events and hazard rations for the explored outcomes in the propensity score matched population according to ejection fraction subtype.

| **Outcome** | **Number of events**  **(per 100 person-years)** | | **HR** | **95% CI** | **p-value** |
| --- | --- | --- | --- | --- | --- |
| **HFrEF** | **Treated**  **N = 417** | **Untreated**  **N = 430** |  |  |  |
| Primary composite | 16.4 | 20.9 | 0.80 | 0.63-1.03 | 0.08 |
| HF hospitalization | 15.1 | 18.8 | 0.83 | 0.64-1.07 | 0.14 |
| CV death | 3.0 | 4.7 | 0.65 | 0.40-1.06 | 0.08 |
| MACE | 5.5 | 8.3 | 0.68 | 0.47-0.98 | 0.040 |
| Stroke/TIA | 1.7 | 1.8 | 0.94 | 0.46-1.90 | 0.86 |
| Myocardial infarction | 1.4 | 2.0 | 0.70 | 0.34-1.48 | 0.35 |
| All-cause death | 5.3 | 8.2 | 0.67 | 0.46-0.96 | 0.029 |
| Repeated HF hospitalizations* | - | - | 0.67 | 0.45-1.00 | 0.05 |
| **HFmrEF** | **Treated**  **N = 168** | **Untreated**  **N = 155** |  |  |  |
| Primary composite | 15.0 | 16.8 | 0.88 | 0.58-1.34 | 0.56 |
| HF hospitalization | 11.8 | 12.8 | 0.91 | 0.56-1.45 | 0.68 |
| CV death | 4.3 | 6.4 | 0.70 | 0.35-1.37 | 0.29 |
| MACE | 8.2 | 9.8 | 0.84 | 0.50-1.44 | 0.53 |
| Stroke/TIA | 2.9 | 1.9 | 1.50 | 0.53-4.21 | 0.44 |
| Myocardial infarction | 2.2 | 2.2 | 0.98 | 0.34-2.78 | 0.96 |
| All-cause death | 6.8 | 9.8 | 0.71 | 0.41-1.23 | 0.23 |
| Repeated HF hospitalizations* | - | - | 1.10 | 0.62-1.97 | 0.75 |
| **HFpEF** | **Treated**  **N = 121** | **Untreated**  **N = 121** |  |  |  |
| Primary composite | 15.0 | 16.0 | 0.93 | 0.57-1.53 | 0.78 |
| HF hospitalization | 14.5 | 13.6 | 1.05 | 0.63-1.77 | 0.84 |
| CV death | 2.5 | 4.4 | 0.57 | 0.21-1.55 | 0.27 |
| MACE | 4.7 | 13.2 | 0.36 | 0.18-0.72 | 0.004 |
| Stroke/TIA | 2.6 | 3.7 | 0.70 | 0.25-1.96 | 0.49 |
| Myocardial infarction | 0.4 | 5.1 | 0.08 | 0.01-0.65 | 0.017 |
| All-cause death | 6.3 | 11.5 | 0.54 | 0.29-1.00 | 0.052 |
| Repeated HF hospitalizations* | - | - | 1.25 | 0.60-2.64 | 0.55 |

P-value for interaction between GLP-1 RA use and EF class for each outcome:

Primary composite: 0.84; HF hospitalization: 0.70; CV death: 0.95; MACE: 0.15; Stroke/TIA: 0.58; myocardial infarction: 0.11; all-cause death: 0.78.

* For repeated HF hospitalization, the point estimate is the incidence rate ratio.

**Legend**: GLP-1 RA, Glucagon-like peptide-1 receptor agonists; HR, Hazard Ratio; CI, Confidence Interval; HFrEF, heart failure with reduced ejection fraction; HFmrEF, heart failure with mildly reduced ejection fraction; HFpEF, heart failure with preserved ejection fraction; HF, Heart Failure; CV, Cardiovascular; MACE, Major Adverse Cardiovascular Events; TIA, Transient Ischemic Attack; EF, ejection fraction.

**Supplemental table 5.** Number of events and hazard ratios, with 95% confidence intervals, in the subgroup of overweight patients in the propensity score matched cohort.

| **Outcome** | **Number of events in the PS matched cohort**  **(per 100 person-years)** | | **HR (95% CI) in the PS matched cohort** | **p-value** | **HR (95% CI) in the PS-adjusted analysis** | **p-value** |
| --- | --- | --- | --- | --- | --- | --- |
| **BMI**$\boldsymbol{\geq}$**25 kg/m^2^** | **Treated**  **N = 658** | **Untreated**  **N = 619** |  |  |  |  |
| Primary composite | 15.7 | 18.5 | 0.86 (0.70-1.04) | 0.12 | 0.86 (0.74-1.01) | 0.07 |
| HF hospitalization | 14.1 | 16.0 | 0.89 (0.72-1.10) | 0.27 | 0.91 (0.76-1.07) | 0.25 |
| CV death | 3.2 | 4.8 | 0.66 (0.45-0.97) | 0.034 | 0.69 (0.51-0.95) | 0.022 |
| MACE | 5.8 | 9.4 | 0.62 (0.46-0.83) | 0.001 | 0.71 (0.56-0.91) | 0.006 |
| Stroke/TIA | 2.0 | 2.3 | 0.88 (0.52-1.47) | 0.62 | 1.08 (0.71-1.63) | 0.72 |
| Myocardial infarction | 1.2 | 2.6 | 0.48 (0.27-0.87) | 0.015 | 0.56 (0.34-0.92) | 0.023 |
| All-cause death | 5.6 | 9.0 | 0.62 (0.46-0.83) | 0.001 | 0.61 (0.48-0.77) | <0.001 |
| Repeated HF hospitalizations* |  |  | 0.88 (0.63-1.22) | 0.43 | 0.83 (0.67-1.03) | 0.09 |

**Legend:** PS, Propensity Score; HR, Hazard Ratio; CI, Confidence Interval; BMI, Body Mass Index; HF, Heart Failure; CV, Cardiovascular; MACE, Major Adverse Cardiovascular Events; TIA, transient Ischemic Attack.

*For repeated HF hospitalizations results are expressed as incidence rate ratio

**Supplemental Table 6**. Number of events in the propensity score matched population and hazard ratios (95% confidence intervals) in the propensity score matched population and in the propensity score-adjusted analysis in the subgroup of patients with obesity, also according to ejection fraction.

| **Outcome** | **Number of events in the PS matched cohort**  **(per 100 person-years)** | | **HR (95% CI) in the PS matched cohort** | **p-value** | **HR (95% CI) in the PS-adjusted analysis** | **p-value** |
| --- | --- | --- | --- | --- | --- | --- |
| **BMI**$\boldsymbol{\geq}$**30 kg/m^2^** | **Treated**  **N = 423** | **Untreated**  **N = 420** |  |  |  |  |
| Primary composite | 14.5 | 20.6 | 0.72 (0.56-0.92) | 0.01 | 0.76 (0.62-0.93) | 0.008 |
| HF hospitalization | 12.9 | 18.2 | 0.73 (0.56-0.95) | 0.02 | 0.78 (0.62-0.96) | 0.023 |
| CV death | 2.8 | 4.8 | 0.58 (0.35-0.94) | 0.03 | 0.60 (0.40-0.91) | 0.016 |
| MACE | 5.1 | 9.2 | 0.56 (0.39-0.81) | 0.002 | 0.63 (0.46-0.85) | 0.003 |
| Stroke/TIA | 1.7 | 1.9 | 0.95 (0.47-1.89) | 0.88 | 0.99 (0.57-1.71) | 0.96 |
| Myocardial infarction | 1.2 | 2.5 | 0.49 (0.24-1.02) | 0.06 | 0.54 (0.29-0.99) | 0.047 |
| All-cause death | 4.9 | 9.6 | 0.51 (0.36-0.73) | <0.001 | 0.51 (0.37-0.69) | < 0.001 |
| Repeated HF hospitalizations* | - | - | 0.77 (0.54-1.09) | 0.14 | 0.76 (0.59-0.98) | 0.037 |
| **According to EF** | | | | | |  |
| **HFrEF** | **Treated**  **N = 237** | **Untreated**  **N = 246** |  |  |  |  |
| Primary composite | 14.7 | 19.3 | 0.77 (0.56-1.08) | 0.14 | 0.77 (0.59-1.01) | 0.06 |
| HF hospitalization | 13.4 | 17.7 | 0.78 (0.55-1.09) | 0.15 | 0.80 (0.60-1.05) | 0.11 |
| CV death | 2.7 | 3.8 | 0.71 (0.36-1.38) | 0.31 | 0.59 (0.35-1.02) | 0.06 |
| MACE | 4.6 | 7.7 | 0.60 (0.37-1.00) | 0.05 | 0.58 (0.38-0.87) | 0.009 |
| Stroke/TIA | 1.1 | 1.7 | 0.62 (0.22-1.77) | 0.38 | 0.62 (0.26-1.46) | 0.27 |
| Myocardial infarction | 1.1 | 2.3 | 0.48 (0.18-1.29) | 0.15 | 0.55 (0.25-1.23) | 0.15 |
| All-cause death | 3.9 | 8.1 | 0.48 (0.29-0.80) | 0.01 | 0.44 (0.29-0.69) | < 0.001 |
| Repeated HF hospitalizations* | - | - | 0.78 (0.50-1.20) | 0.26 | 0.75 (0.54-1.05) | 0.10 |
| **HFmrEF** | **Treated**  **N = 102** | **Untreated**  **N = 90** |  |  |  |  |
| Primary composite | 14.1 | 22.9 | 0.62 (0.37-1.04) | 0.07 | 0.78 (0.52-1.19) | 0.25 |
| HF hospitalization | 11.3 | 18.9 | 0.61 (0.34-1.08) | 0.09 | 0.73 (0.46-1.16) | 0.18 |
| CV death | 3.8 | 6.9 | 0.55 (0.23-1.34) | 0.19 | 0.82 (0.39-1.70) | 0.59 |
| MACE | 7.3 | 10.2 | 0.71 (0.35-1.43) | 0.34 | 0.90 (0.52-1.58) | 0.72 |
| Stroke/TIA | 2.5 | 1.1 | 2.29 (0.44-11.79) | 0.32 | 1.73 (0.65-4.62) | 0.27 |
| Myocardial infarction | 2.0 | 1.7 | 1.18 (0.26-5.27) | 0.83 | 0.76 (0.27-2.18) | 0.61 |
| All-cause death | 6.7 | 10.7 | 0.63 (0.32-1.25) | 0.19 | 0.71 (0.41-1.22) | 0.21 |
| Repeated HF hospitalizations* | - | - | 0.71 (0.40-1.27) | 0.25 | 0.64 (0.37-1.09) |  |
| **HFpEF** | **Treated**  **N = 84** | **Untreated**  **N = 84** |  |  |  |  |
| Primary composite | 14.2 | 22.3 | 0.65 (0.37-1.16) | 0.15 | 0.69 (0.44-1.09) | 0.11 |
| HF hospitalization | 13.5 | 19.1 | 0.73 (0.40-1.33) | 0.30 | 0.77 (0.48-1.23) | 0.27 |
| CV death | 1.7 | 5.6 | 0.30 (0.08-1.13) | 0.08 | 0.37 (0.12-1.18) | 0.09 |
| MACE | 4.2 | 12.7 | 0.33 (0.14-0.78) | 0.01 | 0.49 (0.22-1.04) | 0.06 |
| Stroke/TIA | 3.0 | 3.1 | 0.97 (0.28-3.36) | 0.96 | 1.31 (0.52-3.22) | 0.57 |
| Myocardial infarction | 0.6 | 3.9 | 0.15 (0.02-1.24) | 0.08 | 0.23 (0.03-1.71) | 0.15 |
| All-cause death | 5.8 | 13.0 | 0.45 (0.21-0.95) | 0.04 | 0.49 (0.26-0.92) | 0.026 |
| Repeated HF hospitalizations* | - | - | 0.81 (0.32-2.07) | 0.66 | 0.93 (0.54-1.60) | 0.79 |

P-value for interaction between GLP-1 RA use and EF class for each outcome in the PS matched cohort:

Primary composite: 0.71; HF hospitalization: 0.75; CV death: 0.54; MACE: 0.38; Stroke/TIA: 0.42; myocardial infarction: 0.28; all-cause death: 0.77; repeated HF hospitalizations 0.97.

P-value for interaction between GLP-1 RA use and EF class for each outcome in the PS adjusted cohort:

Primary composite: 0.91; HF hospitalization: 0.95; CV death: 0.51; MACE: 0.32; Stroke/TIA: 0.25; myocardial infarction: 0.58; all-cause death: 0.41; repeated HF hospitalizations 0.62.

* For repeated HF hospitalization, the point estimate is the incidence rate ratio.

**Legend:** PS, propensity score; BMI, body mass index; GLP-1 RA, Glucagon-like peptide-1 receptor agonists; HR, Hazard Ratio; CI, Confidence Interval; HFrEF, heart failure with reduced ejection fraction; HFmrEF, heart failure with mildly reduced ejection fraction; HFpEF, heart failure with preserved ejection fraction; HF, Heart Failure; CV, Cardiovascular; MACE, Major Adverse Cardiovascular Events; TIA, Transient Ischemic Attack; EF, ejection fraction.

**Supplemental table 7.** Outcomes according to age in the PS-matched and PS-adjusted population. Number of events are reported as number per 100 person-years and outcomes are expressed as hazard ratio and 95% confidence interval.

| **Outcome** | **Number of events in the PS matched cohort**  **(per 100 person-years)** | | **HR (95% CI) in the PS matched cohort** | **p-value** | **HR (95% CI) in the PS-adjusted analysis** | **p-value** |
| --- | --- | --- | --- | --- | --- | --- |
| **Age**$\boldsymbol{\geq}$**75 years** | **Treated**  **N = 197** | **Untreated**  **N = 186** |  |  |  |  |
| Primary composite | 22.2 | 22.5 | 0.95 (0.66-1.36) | 0.77 | 0.87 (0.66-1.13) | 0.29 |
| HF hospitalization | 19.9 | 17.4 | 1.10 (0.75-1.60) | 0.63 | 0.95 (0.72-1.27) | 0.75 |
| CV death | 5.5 | 9.0 | 0.61 (0.34-1.11) | 0.11 | 0.65 (0.40-1.05) | 0.08 |
| MACE | 9.1 | 16.5 | 0.56 (0.35-0.89) | 0.013 | 0.69 (0.47-1.01) | 0.06 |
| Stroke/TIA | 4.3 | 3.6 | 1.22 (0.56-2.65) | 0.62 | 1.39 (0.79-2.44) | 0.25 |
| Myocardial infarction | 1.6 | 4.0 | 0.41 (0.14-1.18) | 0.10 | 0.55 (0.23-1.33) | 0.19 |
| All-cause death | 11.5 | 16.5 | 0.71 (0.47-1.07) | 0.10 | 0.69 (0.50-0.97) | 0.031 |
| Repeated HF hospitalizations* | - | - | 1.10 (0.57-2.12) | 0.77 | 0.94 (0.65-1.37) | 0.75 |
| **According to EF** | | | | | |  |
| **Age<75 years** | **Treated**  **N = 509** | **Untreated**  **N = 520** |  |  |  |  |
| Primary composite | 14.1 | 17.9 | 0.80 (0.64-1.01) | 0.06 | 0.87 (0.72-1.05) | 0.14 |
| HF hospitalization | 12.7 | 16.2 | 0.80 (0.63-1.02) | 0.07 | 0.89 (0.73-1.09) | 0.26 |
| CV death | 2.6 | 3.9 | 0.67 (0.42-1.07) | 0.09 | 0.72 (0.49-1.06) | 0.10 |
| MACE | 5.1 | 7.5 | 0.69 (0.48-0.97) | 0.034 | 0.76 (0.57-1.01) | 0.06 |
| Stroke/TIA | 1.5 | 1.8 | 0.85 (0.44-1.63) | 0.62 | 0.98 (0.58-1.66) | 0.94 |
| Myocardial infarction | 1.3 | 2.1 | 0.63 (0.32-1.21) | 0.16 | 0.65 (0.38-1.13) | 0.13 |
| All-cause death | 4.2 | 7.0 | 0.60 (0.42-0.87) | 0.007 | 0.57 (0.43-0.77) | <0.001 |
| Repeated HF hospitalizations* | - | - | 0.71 (0.49-1.03) | 0.07 | 0.79 (0.62-1.01) | 0.06 |

**Legend:** PS, Propensity Score; HR, Hazard Ratio; CI, Confidence Interval; BMI, Body Mass Index; HF, Heart Failure; CV, Cardiovascular; MACE, Major Adverse Cardiovascular Events; TIA, transient Ischemic Attack.

*For repeated HF hospitalizations outcomes are reported as IRR

p-value for interaction of the PS-matched analysis: Primary composite 0.45; HHF 0.17; CV death 0.83; MACE 0.50; Non-fatal stroke/TIA 0.48; Non-fatal myocardial infarction: 0.52; All-cause death: 0.57; Repeated HF hospitalizations: 0.25.

p-value for interaction of the PS-adjusted analysis: Primary composite 0.99; HHF 0.69; CV death 0.71; MACE 0.68; Non-fatal stroke/TIA 0.36; Non-fatal myocardial infarction: 0.74; All-cause death: 0.39; Repeated HF hospitalizations: 0.45.

**Supplemental figure 1.** Percentage of GLP-1 RA users for each year of enrollment.

**Supplemental Figure 2**. HF- and diabetes-related predictors of GLP1-RA use/non-use within the EF subtypes.


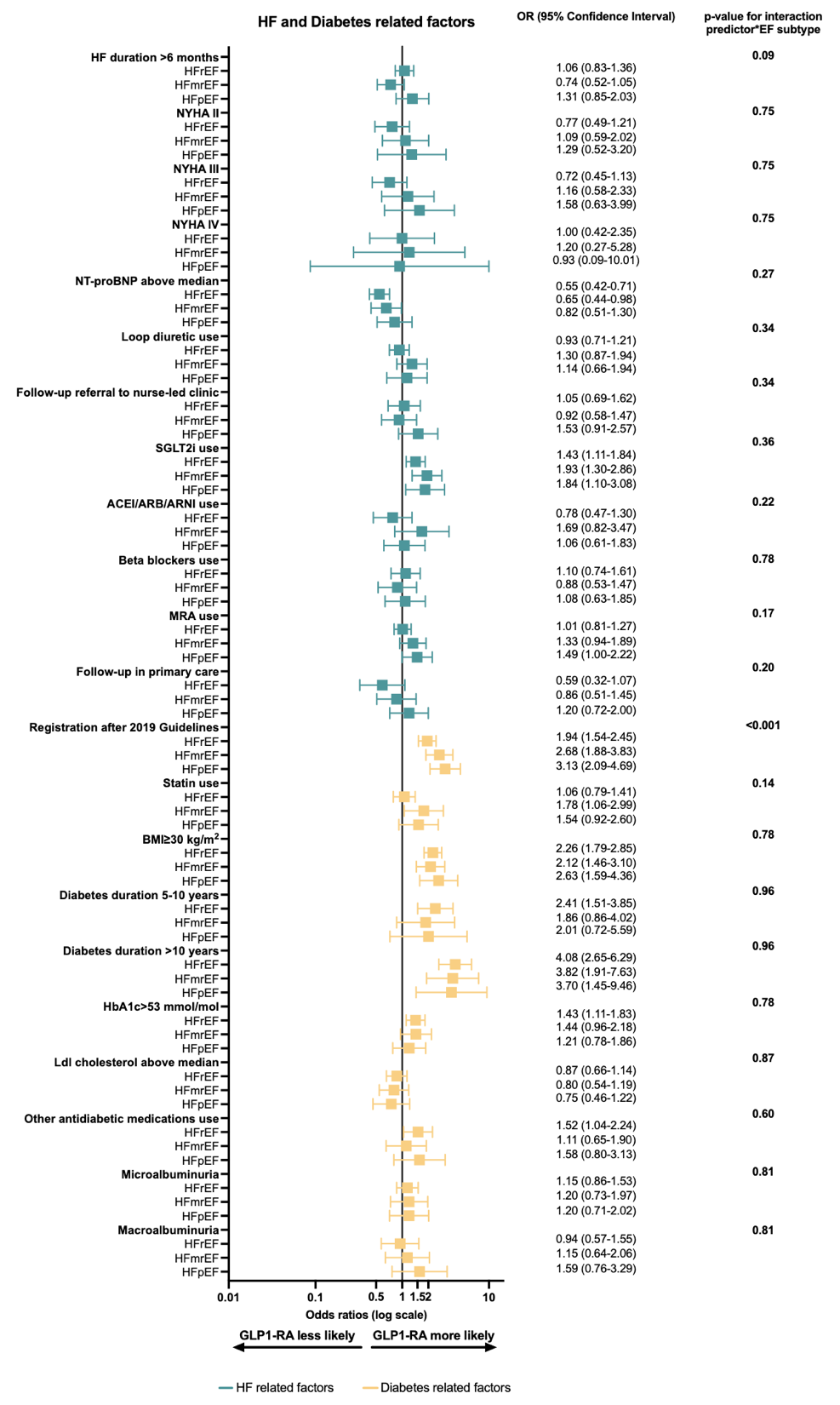


**Legend**: OR, Odds Ratio; BMI, Body Mass Index; SGLT2i, Sodium-Glucose Cotransporter 2 Inhibitors; HbA1c, glycated hemoglobin; MRA, Mineralocorticoid Receptor Antagonists; ACEI/ARB/ARNI, Angiotensin Converting Enzyme Inhibitors/Angiotensin Receptor Blockers/Angiotensin Receptor Neprilysin Inhibitors; NYHA, New York Heart Association; HFmrEF, Heart Failure with Mildly Reduced Ejection Fraction; HFrEF, Heart Failure with Reduced Ejection Fraction; HFpEF, Heart failure with Preserved Ejection Fraction; NT-proBNP, N-Terminal pro-B type Natriuretic Peptide.

**Supplemental Figure 3.** Comorbidities and socioeconomic factors predictive of GLP1-RA use/non-use within the EF subtypes.


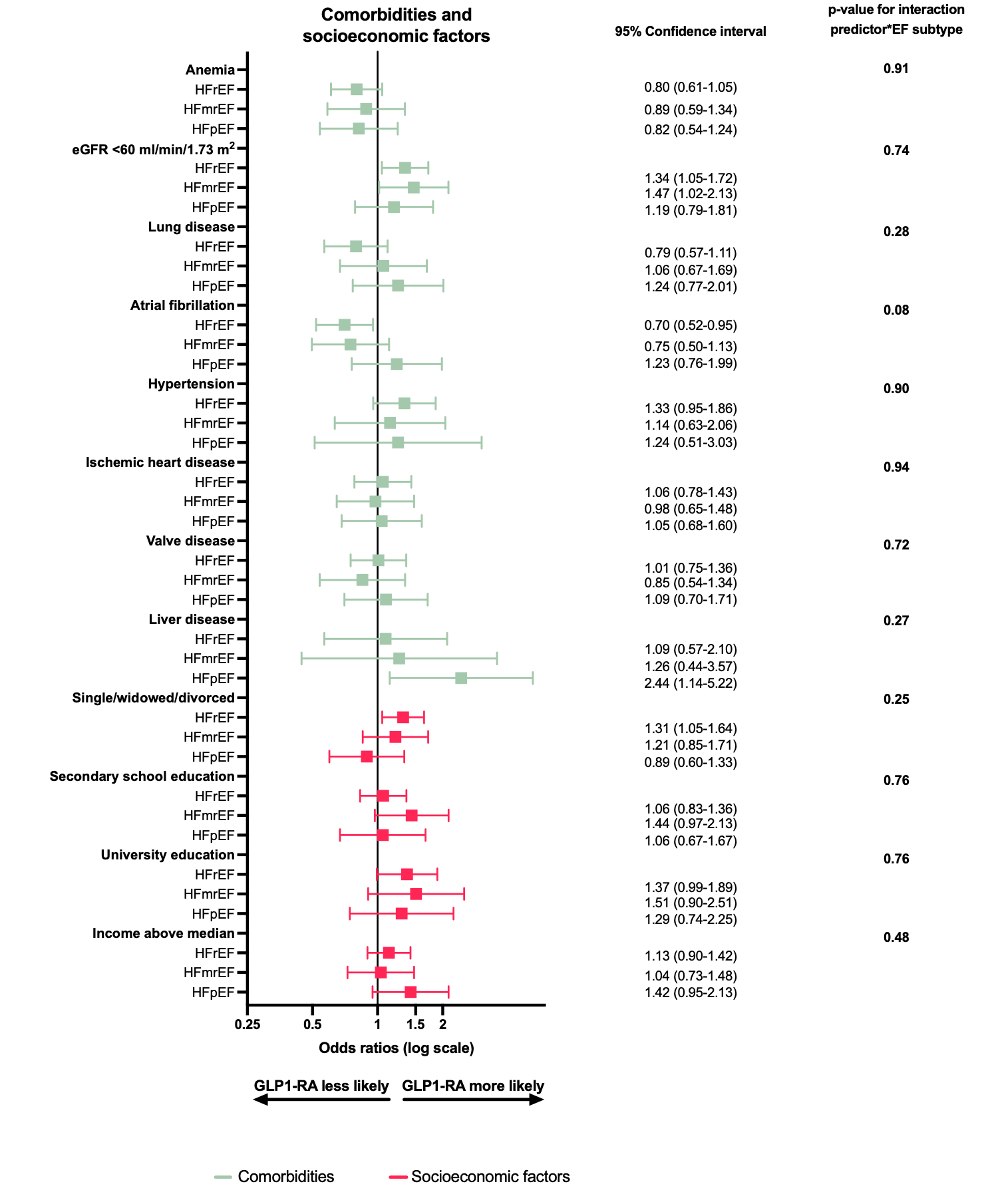


**Legend**: OR, Odds Ratio; eGFR, estimated Glomerular Filtration Rate; HFmrEF, Heart Failure with Mildly Reduced Ejection Fraction; HFrEF, Heart Failure with Reduced Ejection Fraction; HFpEF, Heart failure with Preserved Ejection Fraction.

**Supplemental Figure 4.** Other clinical predictors of GLP1-RA use/non-use within the EF subtypes.

**
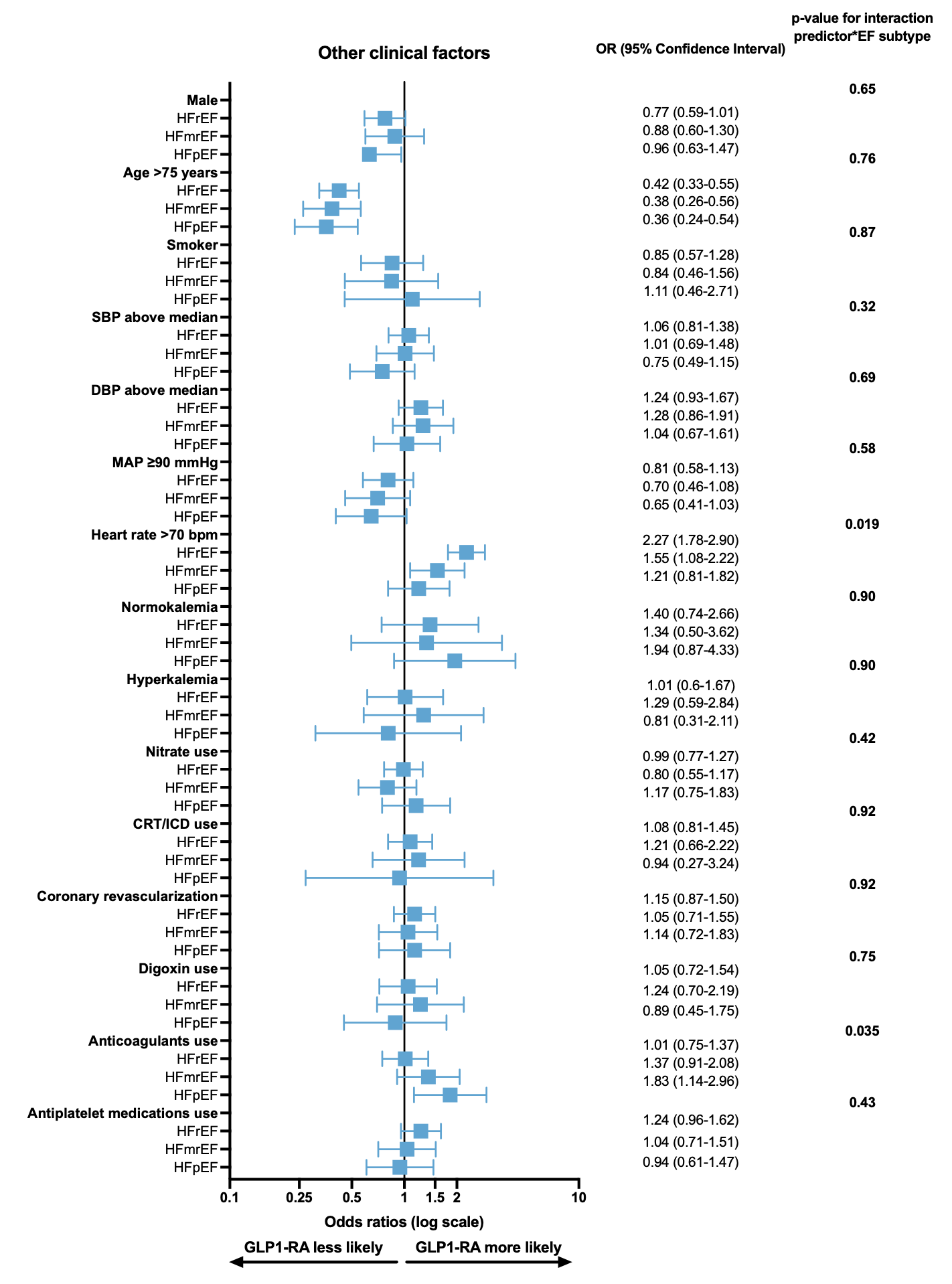
**

**Legend**: OR, Odds Ratio; DBP, Diastolic Blood Pressure; CRT/ICD, Cardiac Resynchronization Therapy/Implantable Cardioverter Defibrillator; SBP, Systolic Blood Pressure; HFmrEF, Heart Failure with Mildly Reduced Ejection Fraction; HFrEF, Heart Failure with Reduced Ejection Fraction; MAP, Mean Arterial Pressure; HFpEF, Heart failure with Preserved Ejection Fraction.

**Supplemental figure 5.** Kaplan-Meier curves displaying outcomes in the propensity score matched cohort.

**Legend:** HF, Heart Failure; CV, Cardiovascular; MACE, Major Adverse Cardiovascular Events; TIA, Transient ischemic Attack; GLP1-RA, Glucagon-like Peptide 1 Receptor Agonists; HR, Hazard Ratio; CI, Confidence Interval.

**Supplemental figure 6.** Outcomes across the EF subtypes in the propensity score matched cohort.

**
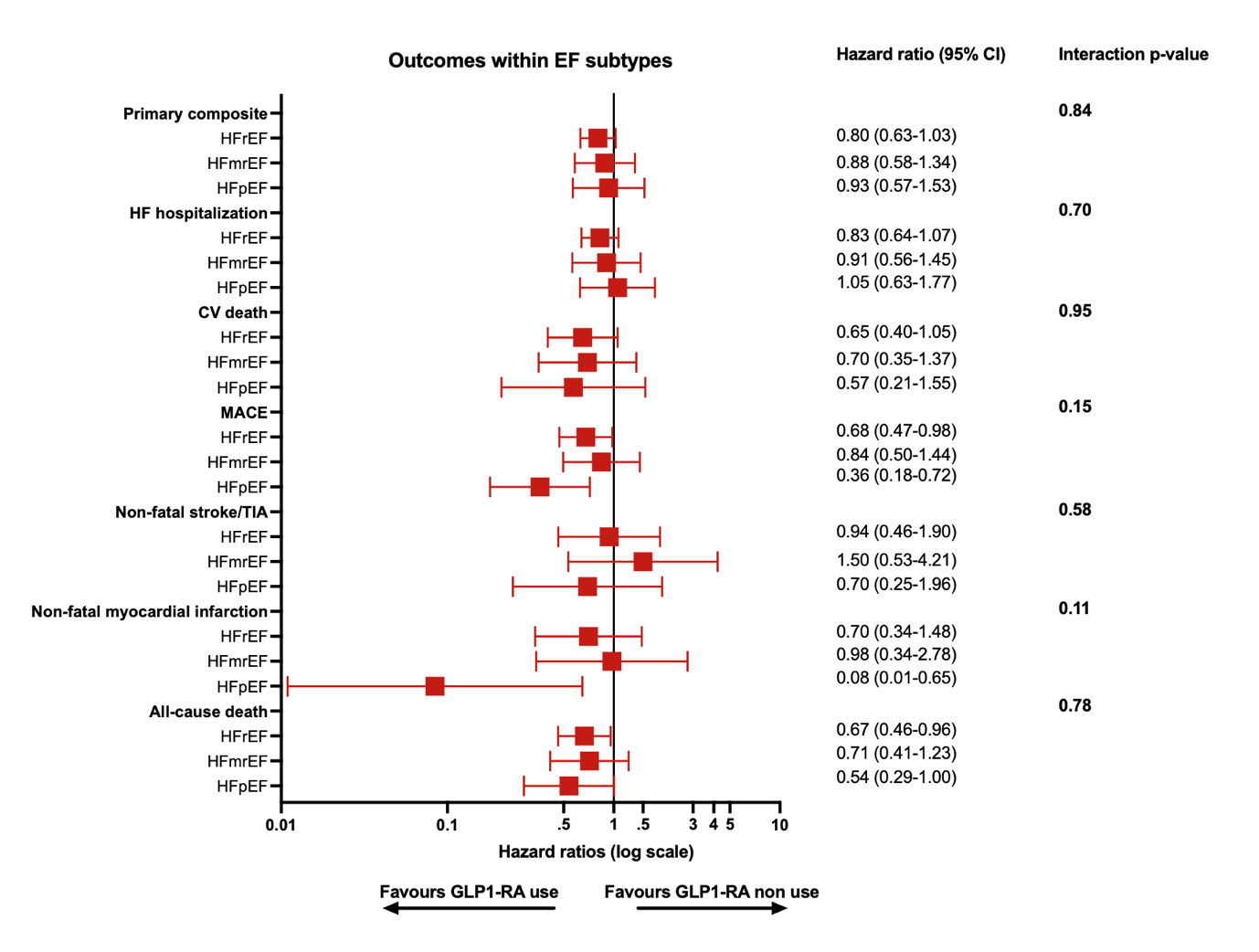
**

**Legend:** EF, Ejection Fraction; CI, Confidence Interval; HFrEF, Heart Failure with reduced Ejection Fraction; HFmrEF, Heart Failure with mildly reduced Ejection Fraction; HFpEF, Heart Failure with preserved Ejection Fraction; HF, Heart Failure; CV, Cardiovascular; MACE, Major Adverse Cardiovascular Events; TIA, Transient Ischemic Attack.
